# Fabrication of ZIF-71/Fe_3_O_4_/polythionine nanoarray-functionalized carbon cotton cloth for simultaneous extraction and quantitation of febuxostat and diclofenac

**DOI:** 10.1039/d1ra04670e

**Published:** 2021-09-13

**Authors:** Yasaman Sanaei, Mohsen Zeeb, Seyed Saied Homami, Amirhossein Monzavi, Zahra Khodadadi

**Affiliations:** Department of Applied Chemistry, Faculty of Science, Islamic Azad University, South Tehran Branch Tehran Iran zeeb.mohsen@gmail.com +98 21 33722831; Department of Polymer and Textile Engineering, South Tehran Branch, Islamic Azad University Tehran Iran

## Abstract

A sustainable hybrid material based on carbonized cotton cloth/zeolite imidazolate framework-71/Fe_3_O_4_/polythionine (CC/ZIF-71/Fe_3_O_4_/PTh) was synthesized and applied in ultrasound-assisted dispersive magnetic solid-phase extraction (USA-DMSPE) followed with high performance liquid chromatography-ultraviolet detection (HPLC-UV) for simultaneous quantitation of diclofenac (DIC) and febuxostat (FEB) in human plasma. The surface of CC was modified with nanoarrays of zeolite imidazolate framework-71/Fe_3_O_4_/Polythionine. At first, an *in situ* synthesis of ZIF-71 in the presence of CC was carried out, and followed with magnetization process and oxidative polymerization of thionine. The nano-modifier agents improved the merits of the sorbent involving stability, porosity, mast transfer, π–π interactions and selectivity of CC. Characterizations of the hybrid sorbent were examined with different instrumental techniques. The limits of detection (LODs, *S*/*N* = 3) were recognized 2.1 ng mL^−1^ for DIC and 3.7 ng mL^−1^ for FEB. Acceptable linearity (0.992 ≤ *r*^2^ ≤0.996) and relatively broad dynamic ranges of 10.0–1800.0 ng mL^−1^ and 15.0–2500.0 ng mL^−1^ were achieved for DIC and FEB, respectively. Reasonable intra-assay (≤7.2%, *n* = 9) and inter-assay (≤7.5%, *n* = 9) precisions as well as appropriate accuracies (≤8.0%) were provided illustrating applicability of the current approach for analytical purposes. Eventually, CC/ZIF-71/Fe_3_O_4_/PTh was employed as four-part sorbent for the assessment of DIC and FEB in human plasma at trace levels and subsequently main pharmacokinetic data such as *T*_1/2_, *T*_max,_*C*_max_, and AUC_0–24_ of these drugs were comprehensively investigated.

## Abstract 2

Synthesis of a material based on carbonized cotton cloth/zeolite imidazolate framework was applied to ultrasound-assisted dispersive magnetic solid-phase extraction and high-performance liquid chromatography-ultraviolet to detect diclofenac and febuxostat in human plasma.

## Graphical abstract

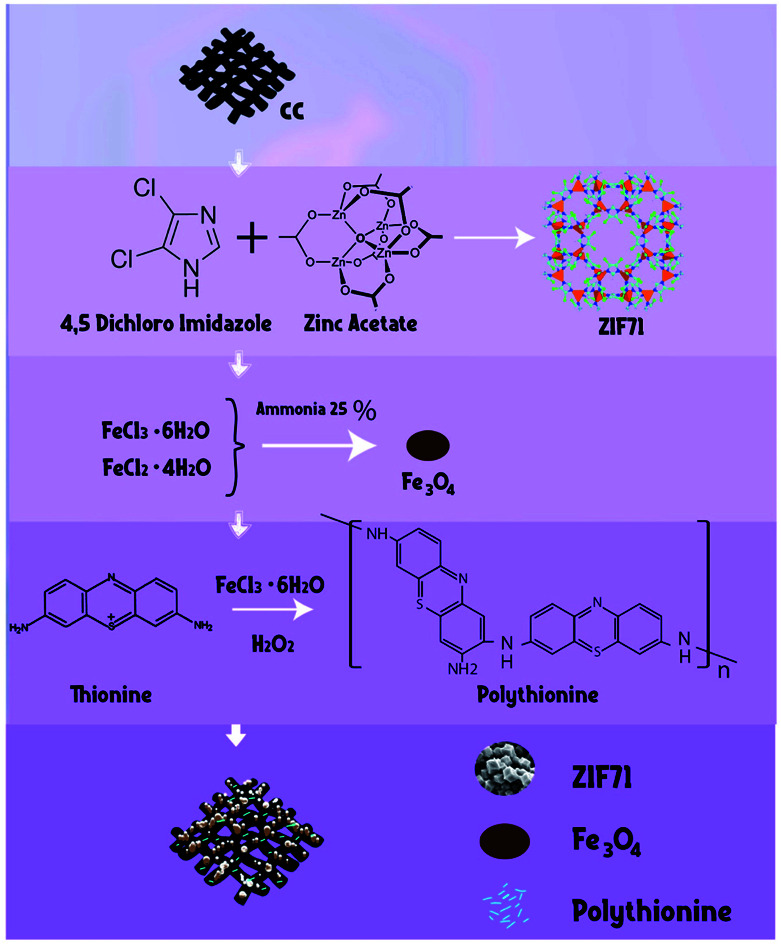

## 1. Introduction

Increase in concentration level of uric acid in serum causes gout which is a common arthritic disease. In order to treat gout, non-purine xanthine oxidase inhibitors such as febuxostat 2-(3-cyano-4-isobutoxyphenyl)-4-methyl-1,3-thiazole-5-carboxylic acid (FEB) are widely used to decrease the uric acid content in human serum. Furthermore, non-steroidal anti-inflammatory drugs (NSAIDs) like diclofenac 2-(2,6-dichloranilino)phenylacetic acid (DIC) are applied for patients to manage inflammation and pain associated with joint disorders.^1,2^ DIC was utilized in combination with FEB to benefit from the properties of both drugs for curing and controlling all problems associated with gout illness along with an increase in terms of convenience and patient compliance. Therapeutic drug assessment is important to confirm the quantity of plasma content for pharmacokinetic investigations, bioequivalence valuation of tablet formulation, optimization of new and practical dosage forms and dosing regimen in combination therapy for diagnostic aims.^3–6^

Literature survey exhibits that different analytical protocols involving gas chromatography, high performance liquid chromatography followed with mass spectrometry (HPLC-MS/MS), RP-HPLC with ultraviolet and fluorescence detections, high-performance thin-layer chromatography (HPTLC) and spectrophotometric methods have been reported for the determination of FEB and DIC in real samples.^7–17^

Recently, dispersive magnetic solid-phase extraction has been developed as a new and practical kind of solid-phase extraction (SPE), which benefit from some merits such as reduction of hazardous solvent usage, elimination of time consuming routes like filtration and centrifugation, acceptable enrichment values along with reasonable removal of interfering species.^18–20^ This enrichment protocol has been widely used to analyze environmental, biological, pharmaceutical and food samples with satisfactory results.^19–23^ Extreme attempts have been conducted to introduce new magnetic sorbents and apply them in special analytical purposes.^24–27^ However, the introduced extractors suffer from some disadvantages such as lack of reusability, low surface area-to-volume ratio, insufficient porosity, high cost of synthesis, unacceptable selectivity, *etc.* In this regard, carbonized cotton cloth (CC) as a novel class of carbon-based material, seems superior substance for fabricating an efficient and reusable hybrid material for extraction and enrichment of target compounds form complex matrices.^28^ CC can be obtained from cotton, linen, cloth or felt which are easily available, and furthermore it benefits from some features including low cost of fabrication, simple synthesis route, considerable extraction ability along with properties which correspond to the concept of green chemistry.^29,30^

In recent years, in order to increase the merit of CC, its surface has been functionalized with different materials including Fe_3_O_4_,^31^ SnO_2_,^32^ Ni(OH)_2_ nanostructure,^33^ zinc sulfide/copper sulfide,^34^ ZnO/metal–organic framework/polyaniline,^35^ metal–organic framework/sulfonated polythiophene.^36^ Among these modifier agents, metal–organic frameworks (MOFs) as 3-dimensional structures exhibit specific features like high surface area from 1000 to 10 400 m^2^ g^−1^, adequate resistance, tunable porosity and simple synthesis routes. As a result, MOFs are appropriate options for fabricating hybrid substances with notable extraction and dispersion capability as well as reasonable mechanical strength.^35–38^ Zeolitic imidazolate frameworks (ZIFs) are classified as a novel type of crystalline MOFs which can be prepared *via* coordination between metal ions (clusters) and imidazole/imidazolate as organic linkers.^39^ The combination of CC and ZIFs significantly increases the merits of sorbent and makes this carbon-based material a promising sorbent for sample preparation goals.^40–42^ ZIF-71 is one part of the great class of ZIFs, which can be fabricated at room temperature. In addition, ZIF-71 offers many advantages including low required synthesis temperature, short preparation time, simple synthesis rout, low cost, *etc.*^43–45^ Hence, in this work, ZIF-71 was preferred to other MOFs and used as a modifier agent of CC. In order to improve the properties of carbon based material for isolation and enrichment purposes, conductive polymers such as polythionine, polyaniline, polythiophene, polytyramine seem appropriate choices which extensively enhance π–π interaction, hydrophobic possessions, extraction facility, diffusion rate and reusability.^24–27,35,36,46^

In the presented study, CC was functionalized with ZIF-71 to result CC/ZIF-71 and followed with coprecipitation of Fe_3_O_4_ to provide a super-magnetic material. In the next synthesis route, polymerization of thionine was performed on the surface of CC/ZIF-71/Fe_3_O_4_ to finalize the modification of CC with nano-arrays of substances. The new fabricated hybrid material was utilized as recyclable sorbent in USA-DMSPE. FEB and DIC in human plasma were subjected to extraction protocol and followed by HPLC-UV to illustrate the applicability of the fabricated sorbent for analyzing trace level amounts of drugs in biological media. Although the CC, PTh, Fe_3_O_4_ and ZIF-71 has been examined separately and according to the best of our knowledge, any study has not been reported on the novel fabricated hybrid sorbent (CC/ZIF-71/Fe_3_O_4_/PTh) in ultrasound-assisted dispersive magnetic solid-phase extraction (USA-DMSPE) for simultaneous extraction and quantitation of drugs in biological media. The main experimental variables that influence the extraction effectiveness were studied in detail and optimized. Eventually, the possibility of the currently designed sorbent was perused by measuring main pharmacokinetic data of FEB and DIC in human plasma.

## 2. Experimental

### 2.1. Chemicals

In all optimization and measurement steps, analytical grade of each chemical was used without extra purification. These chemicals involving ethanol (C_2_H_5_OH), methanol (CH_3_OH), chloroform, ferric chloride (FeCl_3_·6H_2_O), ferrous chloride (FeCl_2_·4H_2_O), ammonia (NH_3_, 25%), hydrogen peroxide (H_2_O_2_, 30%), thionine (Th) acetate (85%), HPLC grades of methanol, acetonitrile and acetone were purchased from Merck company (Darmstadt, Germany). Zinc acetate, 4,5-dichloroimidazole, phosphoric acid (H_3_PO_4_) 85% were bought from Sigma-Aldrich (Taufkirchen, Germany). Commercial cotton cloth composed of identical 100% cotton yards with a plain weave was supplied from a nearby market. The standards of diclofenac and febuxostat drugs were purchased from Darupakhsh Company (Tehran, Iran). Ultrapure water (Millipore, Bedford, MA, USA) was used in all experiments.

### 2.2. Instrumentation

Fourier transform-infrared (FT-IR) spectra were recorded and studied using A Tensor 27 FT-IR spectrometer (Bruker, Germany). The X-ray diffraction (XRD) spectra were investigated and documented utilizing Cu Kα radiation (*λ* = 1.5406 Å) on X'Pert PRO MPD X-ray diffractometer (PANalytical Company, Netherlands). Field-emission scanning electron microscopy (FE-SEM) were recorded by applying a Mira 3-XMU (Tescan, Czech Republic). Commercial cotton cloth was carbonized using a laboratory tube furnace 1200 °C (Exiton, Iran).

### 2.3. Chromatographic conditions

Chromatographic measurements were performed using a waters alliance e2695 (Massachusetts, USA) coupled to a waters 2487 dual wavelength detector. All separation processes were done on a C_18_ reversed phase column at operating temperature of 30 °C (luna 5 μm C_18_ 100A HPLC column 250 × 4.6 mm id, phenomenex Co, Torrance, CA). The drugs under study were eluted in an isocratic strategy. Methanol-formic acid at pH 2 with volume ratio of 70 : 30 V/V was used as the mobile phase. All the separations were carried out at 1.5 mL min^−1^, which was controlled using the mobile phase pump. The eluting solvent was filtered through a 0.2 μm membrane filter consisting of polytetrafluoroethylene (PTFE) (Millipore, Bedford, MA, USA) also it was degassed every single day prior to separation. UV wavelength and injection volume were fixed at 260 nm and 20 μL, respectively.

### 2.4. Fabrication of CC

Carbonized cotton cloth (CC) was prepared based on a reported method in literature.^47^ The commercial cotton cloth with a tabby weave was cut into squares of 6.4 × 6.4 cm^2^ and it was rinsed by distilled water and non-ionic detergent. After that the solution containing the cotton cloth was stirred for an hour at 60 °C and then it was dried in an oven under vacuum at 80 °C. Chemical activation process of cotton cloth was performed by applying phosphoric acid (H_3_PO_4_). For this purpose, cotton cloth was placed in phosphoric acid with 1.5 impregnation ratio for 24 hours and was dried in an oven under vacuum at 90 °C. In the next route, in order to prepare the desirable CC, the chemical activated cotton cloth was carbonized in a tube oven at operating temperature of 500 °C for 85 min under nitrogen atmosphere at a heating rate of 6 °C min^−1^. The CC was allowed to be cooled overnight at room temperature (23 ± 0.5 °C) and then in order to remove impurities, it was eluted with distilled water several times. Finally, it was placed in an oven under vacuum at 80 °C for 30 min to obtain dried CC.

### 2.5. Fabrication of CC/ZIF-71

ZIF-71 nanocrystals were synthesized according to a previously reported method^44^ as follows: 0.07 g zinc acetate and 0.2 g 4,5-dichloroimidazole were separately dissolved in 15 mL of methanol and then they were mixed together in a sealed sample vial. 0.095 g of the synthesized CC was dipped in the above solution and kept stand at room temperature (23 ± 0.5 °C) for 24 h. After that, in order to omit the solvent, methanol was sucked by using a pipette and the obtained product was soaked in 3 × 20 chloroform for three days. In the final step, the fabricated CC/ZIF-71 was dried in an oven at 90 °C under vacuum for 1 h.

### 2.6. Fabrication of CC/ZIF-71/Fe_3_O_4_

Fe_3_O_4_ nanoparticles were synthesized based on a reported protocol.^25^ Briefly, 0.246 g FeCl_3_·6H_2_O and 0.096 g of FeCl_2_·4H_2_O were dissolved in 22.5 mL distilled water and after that the solution was placed into a three-necked flat bottom flask containing 0.093 g of previously fabricated CC/ZIF-71. In the next step, the resultant solution was stirred for one hour at room temperature (23 ± 0.5 °C). The fabrication route was followed by adjusting the pH of the mixture solution at 10 by adding appropriate amount of ammonia (25% v/v) and then the temperature was moved up to 80 °C and stirred for 1 extra hour. All the steps were performed under nitrogen atmosphere. In the last step, the resulting super magnetic product was separated from the media with an external magnet and washed with distilled water and ethanol four times and afterwards it was followed by drying at 80 °C in an oven under vacuum for 20 min.

### 2.7. Fabrication of CC/ZIF-71/Fe_3_O_4_/PTh

**Fig. 1:**
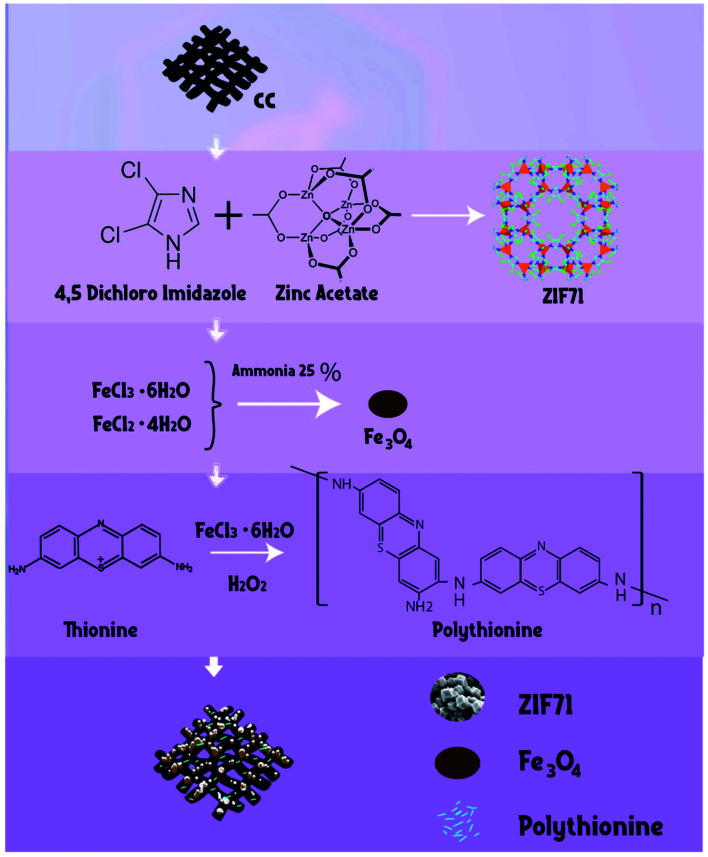
Scheme of CC/ZIF-71/Fe_3_O_4_/PTh preparation stages.

PTh was deposited on the surface of CC/ZIF-71/Fe_3_O_4_ using an oxidative chemical polymerization method as reported previously.^26^ The preparation CC/ZIF-71/Fe_3_O_4_/PTh was started by transferring 0.09 g of CC/ZIF-71/Fe_3_O_4_ in a beaker containing 22.5 mL water. Then, 0.06 g thionine (Th) and a measured amount of FeCl_3_·6H_2_O as catalyst (0.03 g) were dissolved in 12.5 mL water in a round bottom flask and afterwards the obtained solution was added to the reaction mixture with interval time of 2 min. To follow chemical polymerization, 0.6 mL of oxidizing agent (H_2_O_2_) was added to the final prepared mixture gently during stirring. The temperature of mixture was increased to 50 °C and it was stirred for 60 min to vanish the purple color of the media, which shows the successful fabrication of polythionine. Lastly, the resulting product was separated by a strong magnet, eluted with deionized water several times and then dried overnight to obtain the final hybrid material. Schematic diagram of all fabrication routes are shown in Fig. 1.

### 2.8. Standard solutions and quality control samples of diclofenac and febuxostat

Standard stock solutions of DIC and FEB were separately prepared at concentration level of 200.0 mg L^−1^ every week to avoid decomposition of each drug. To prepare these solutions, definite amount of each drug was dissolved in methanol media. Step-wise dilutions were applied with deionized water to fabricate working standard solutions of FEB and DIC. Due to the matrix effect of biological media, human plasma samples were spiked with various volumes of FEB and DIC working solutions to obtain desirable level of standard calibration samples for drawing calibration curve. For further investigations of accuracy and precision of the developed protocol in trace level quantitation of drugs, quality control samples were prepared in three levels of 10.0, 150.0 and 1000.0 ng mL^−1^ for DIC along with 15.0, 150.0 and 1000.0 ng mL^−1^ for FEB. All solutions were stored in a dark place at temperature of −18 °C before quantification.

### 2.9. Deproteinization of human plasma

First of all, some frozen human plasma samples were allowed to be thawed at room temperature (23 ± 0.5 °C) and afterwards they were placed into centrifuge tubes. Acetonitrile was applied as a deproteinizing agent to precipitate all proteins existing in plasma. In this regard, 1.9 mL of plasma was spiked with 100.0 μL of working standard solutions to get the suitable concentration level of DIC and FEB, then, to all tubes, 2.0 mL acetonitrile was added. All the biological samples were vortexed for 3 min for complete mixing and followed with centrifugation at 5000 rpm for 4 min. For evaporating the acetonitrile content of each real sample, the tube containing the spiked sample was placed under a stream of nitrogen. To adjust the pH of media, 4.0 mL buffer solution at pH 5.0 was added to real sample. Eventually, 5.0 mL of the resultant spiked sample was subjected to the extraction and measurement protocols for further evaluations.

### 2.10. The procedure of USA-DMSPE-HPLC-UV

**Fig. 2:**
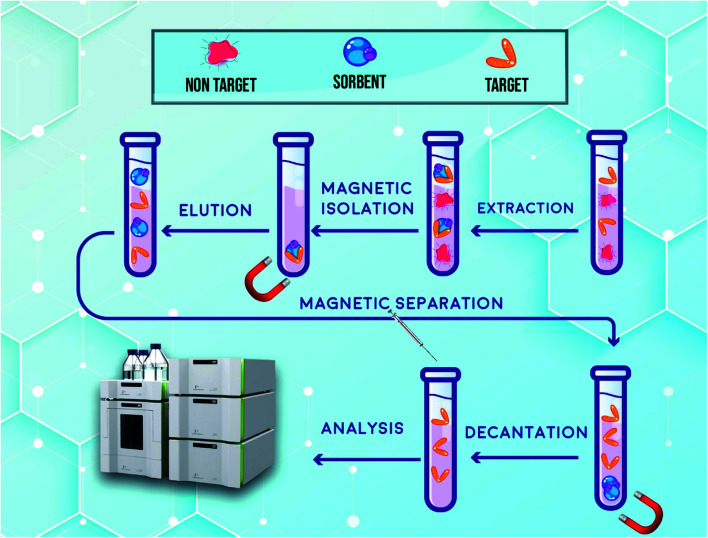
Schematic illustration of all routes of USA-DMSPE-HPLC-UV process for isolation and determination of DIC and FEB.

5.0 mL of the prepared spiked human plasma was placed into a centrifuge tube and then 20.0 mg of synthesized hybrid sorbent was added to the tube. For ensuring the complete dispersion of the supermagnetic hybrid sorbent into the solution, ultrasonic irradiation was utilized for 6 min. As the sorbent is dispersed through the sample, the target drugs are adsorbed on the surface of it. Subsequently, an external magnetic field composed of neodymium (Nd), iron (Fe) and boron (B) (0.8 tesla) was applied to separate the hybrid sorbent from media and the free drug sample was removed by a pipette. In order to desorb the target drugs from the extractor, it was washed with 1.5 mL with acetonitrile in three steps (0.5 mL in each step) while ultrasonic irradiation was applied for 3 min. Nd-Fe-B-Nd magnet was used again in this step and then the solution was separated and dried under a stream of nitrogen. After collecting the remaining material, which contains DIC and FEB, was dissolved in 50.0 μL of the mobile phase of HPLC and 20.0 μL of it was injected into the separation column and further measurements were carried out. All routes of USA-DMSPE-HPLC-UV for extraction, enrichment and measurement of DIC and FEB are shown in Fig. 2.

## 3. Results and discussion

### 3.1. Characterizations

**Fig. 3:**
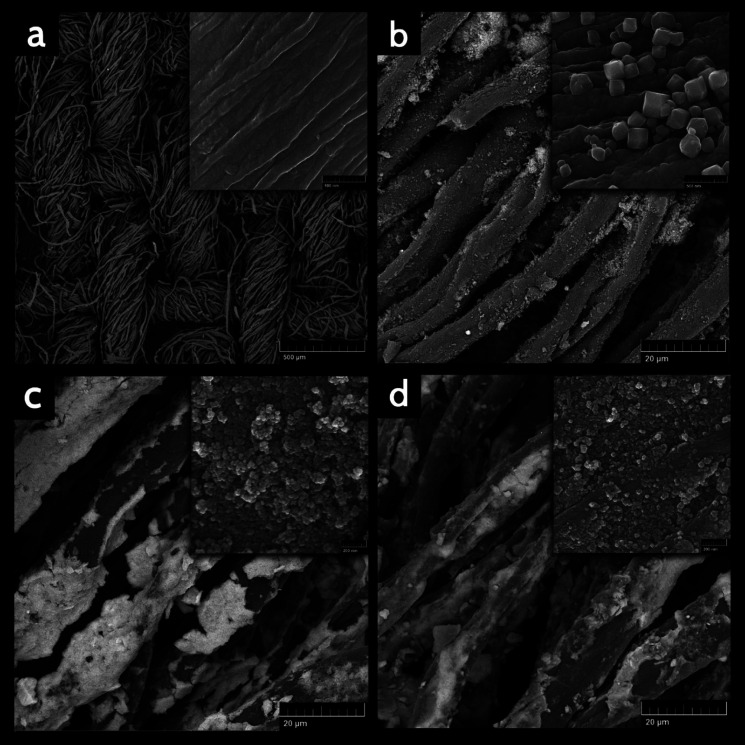
Field-emission scanning electron microscopy (FE-SEM) micrographs of (a) CC (b) CC/ZIF-71 (c) CC/ZIF-71/Fe_3_O_4_ (d) CC/ZIF-71/Fe_3_O_4_/PTh. Inset: magnified view of the nanoarray substances and CC.

Field-emission scanning electron microscopy (FE-SEM) was applied to characterize the surface morphology of synthesized nanohybrid sorbent in this work. As it is shown in Fig. 3a the prepared CC had a plain weave structure, homogeneous and smooth surface morphology. The crystalline structure of ZIF-71 are grown on the surface of CC with size around 100 nm and they can be clearly seen in Fig. 3b. According to the Fig. 3c, the magnetic substance (Fe_3_O_4_ nanoparticles) with the average size of 20 nm were efficiently coated on CC/ZIF-71. As it can be seen in Fig. 3d, CC/ZIF-71/Fe_3_O_4_ were coated with PTh, whereas the size of them has been converted to about 30 nm. A magnified view of the nanoarray substances and CC are inserted into the corresponding images. Evidently, as a consequence of the existence of nanoarrays, which utilized as modifier agents, the surface of CC/ZIF-71, CC/ZIF-71/Fe_3_O_4_ and CC/ZIF-71/Fe_3_O_4_/PTh seem jagged in comparison with the CC.

**Fig. 4:**
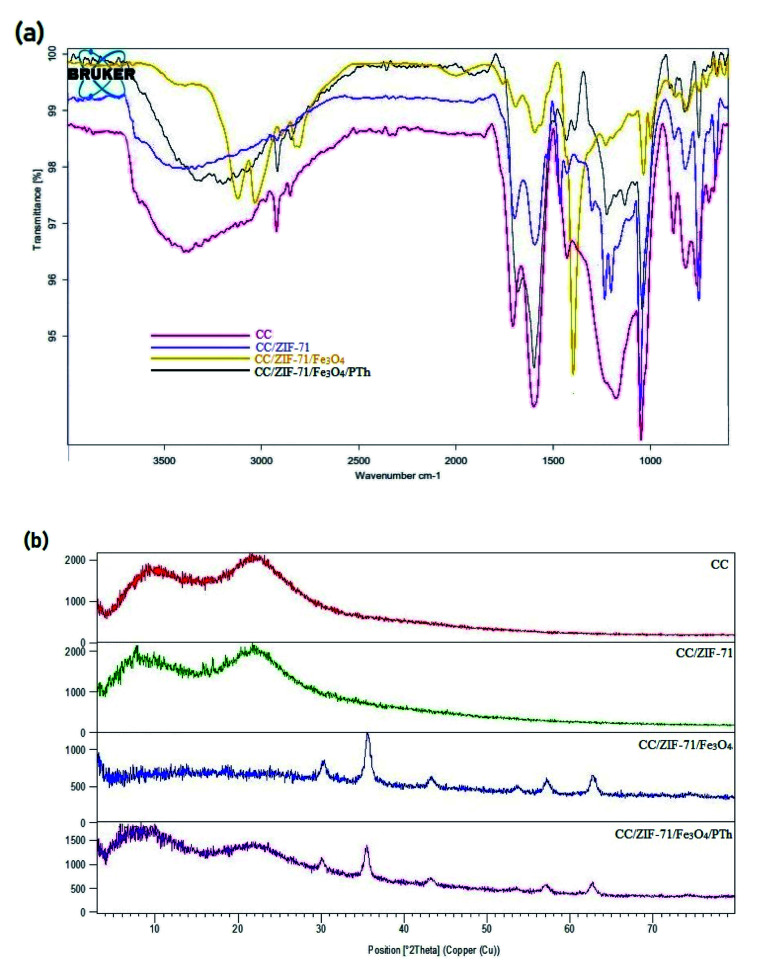
(a) FTIR and (b) XRD spectra of sorbent through all the steps of synthesis.

For more investigation, the chemical structure of CC, CC/ZIF-71, CC/ZIF-71/Fe_3_O_4_ and CC/ZIF-71/Fe_3_O_4_/PTh were characterized by recording FTIR spectra in the range of 400–4000 cm^−1^ (Fig. 4a). The peak at 1702 cm^−1^ assigned to a stretching vibration of the ester group in the CC structure; While, the asymmetric and symmetric stretching vibrations of carboxylate groups are located at 1595 and 1423 cm^−1^, respectively. The peak which corresponded to bending vibrations of methylene (–CH_2_–) was revealed at 755 cm^−1^ as well as the peak at 3393 cm^−1^ was marked to the O–H stretching vibration, in the CC structure. Moreover, FT-IR spectra of the CC/ZIF-71 demonstrate a medium band at 663 cm^−1^ that can be corresponded to the C–Cl stretching vibration of 4,5-dichloroimidazole ligand. The noticeable peaks at 1300, 1594 and 1697 cm^−1^ were assigned to C–N, symmetric C=O, and asymmetric C=O stretches of the imide structure, respectively. Furthermore, the peaks at 1428 and 1464 cm^−1^ attached to N–H bonds of secondary amine, besides the peak at 3342 cm^−1^ were referred to free non-hydrogen bonded N–H bonds of ZIF-71 nanocrystals. Due to the modification of the CC/ZIF-71 by utilizing Fe_3_O_4_-NPs, two peaks were observed at around 623–660 cm^−1^ and 3039 cm^−1^, which attributed to the stretching vibrations of Fe–O. C–N stretching vibrations and aromatic C–H stretching vibrations of polythionine were observed at 1041 and 2918 cm^−1^, respectively. In addition, the peak at 3198 cm^−1^ assigned to the N–H stretching vibration; while, the peaks at 1680 and 1598 cm^−1^ demonstrated that the N–H scissoring of the primary amino moieties existed in the CC/ZIF-71/Fe_3_O_4_/PTh structure. The peaks at 1431 and 800 cm^−1^ were linked to the aromatic C=C stretching vibration and C–S group of polythionine, respectively. The results proved that the CC/ZIF-71/Fe_3_O_4_/PTh was successfully prepared.

For more investigation, the structure of the sorbent in four parts were surveyed by X-ray diffraction (XRD) technique. According to the results in Fig. 4b, the CC spectrum showed the diffraction peaks of graphite carbon at 9° and 22.2° 2*θ*. Additionally, the peaks at about 5° and 15° 2*θ* can be observed which are attributed to nanocrystal structure of ZIF-71 in CC/ZIF-71 structure. After modification of CC/ZIF-71 by pure cubic spinel crystal magnetic sorbent (Fe_3_O_4_-NPs), the CC/ZIF-71/Fe_3_O_4_ spectrum demonstrated seven noticeable peaks at 30.30°, 35.63°, 43.31°, 53.64°, 57.20°, 62.76° and 74.42° 2*θ*. It must also be noted, the pattern of CC/ZIF-71/Fe_3_O_4_/PTh which had the notable peaks of Fe_3_O_4_-NPs were kept unchanged but the considerable diffraction peak for CC was diminished to 2*θ* = 8.2° disclosing the high magnetic feature of sorbent and its suitability for the magnetic separation.

### 3.2. Influence of hybrid sorbent dosage

**Fig. 5:**
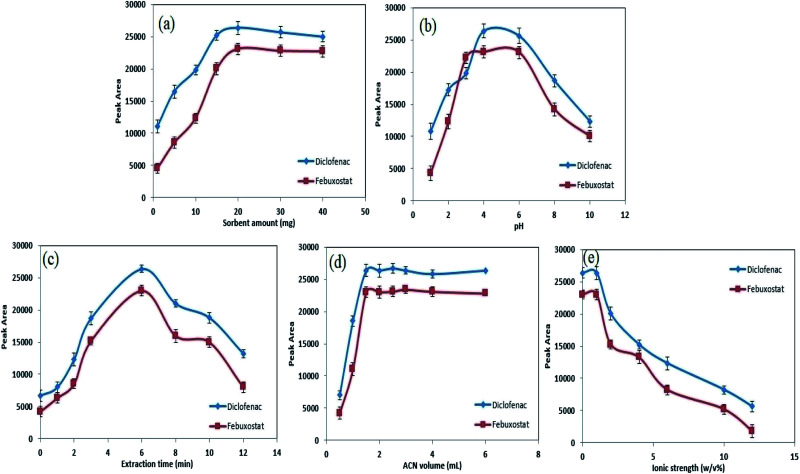
Factors that influence the extraction efficiency. Effect of the amount of nanohybrid sorbent (a), pH (b), extraction time (c), the volume of ACN (d), ionic strength of sample media (e) on the extraction efficiency of DIC and FEB at concentration level of 150.0 ng mL^−1^ for each drug with a sample volume of 5.0 mL.

The dosage of hybrid sorbent in extraction methods is a vital variable which affect enrichment factor, recovery value, sensitivity and reproducibility of data.^24^ Therefore, several amounts of CC/ZIF-71/Fe_3_O_4_/PTh within the range of 1.0–40.0 mg were subjected to developed analytical method, in order to find the optimum extraction condition. The results are shown in Fig. 5a and according to the obtained data, the peak area of DIC and FEB depends on the amount of hybrid sorbent from 2.0 to 20.0 mg. In this regard, by growing the amount of extractor, the analytical signals of analytes increased and the highest sensitivity was obtained in an approximately low amount of sorbent (20.0 mg). At higher values of sorbent, a meaningful decrease in peak area of drugs were observed which is due to this fact that by increasing the amount of extractor, the desorption of analytes from the surface could not be completed. According to these criteria, a value of 20.0 mg of CC/ZIF-71/Fe_3_O_4_/PTh was selected as the optimum dosage amount for the rest of the experiments.

### 3.3. Influence of pH

The pH of the sample can affect the structure of the ionizable compounds like DIC and FEB and lead to form neutral or ionic form of target substances which control their isolation yields form the aqueous media. As DIC and FEB contain carboxylic and amino groups in their structures, p*K*_a_ values must be taken to the account to explain the observed results on the extraction depending on the pH of the sample. The reported p*K*_a_ values for FEB and DIC are 3.3 and 4.15.^48,49^ The influence of the pH on the extraction and peak area of DIC and FEB was investigated in the range of 1.0–10.0 using 0.01 M HCl and NaOH (Fig. 5b). In order to obtain a compromise between sensitivity, reproducibility and simultaneous analysis of target drugs, pH 4.0 seems to be good choice for subsequent experiments. This result can be explained as follows: due to the p*K*a values of DIC and FEB, at acidic pH, neutral forms of these drugs are dominate leading to stronger hydrophobic–hydrophobic interactions between analytes and sorbent, which causes higher analytical responses. So, pH 4.0 was selected as the optimal for the rest of the work.

### 3.4. Influence of extraction time

The time of ultrasonic irradiation is a critical factor and it should be taken to the account as the extraction time.^50^ The impact of the current variable on the peak area of FEB and DIC was surveyed from 0 to 12 min and corresponding results are shown in the Fig. 5c. As it is clear, permanent and satisfactory responses were achieved at value 6 min, which is a relatively low extraction time. However, at values above 6 min, the condition altered and a significant reduction in mass transfer and subsequent analytical signal was seen. The latter happening occurs because of this fact that at higher ultrasonic irradiation times, some parts of drugs are separated from the sorbent and return to the sample media causing a reduction in extraction yield. So, 6 min of applying ultrasonic irradiation is enough to cover all the necessities.

### 3.5. Desorption condition

After adsorbing the drugs on the surface of sorbent and controlling the main variables, precise control on desorption process from drug-loaded sorbent plays a critical role to obtain reliable and stable data. For this goal, some kinds of organic solvents involving methanol, acetonitrile and acetone can perform the desorption of drugs from the surface of hybrid extractor. Hence, the influence of these organic desorbing agents on the enrichment protocol were evaluated in detail. Methanol and acetone revealed poor desorbing ability in comparison with acetonitrile, meanwhile by using acetonitrile a considerable extraction yield along with satisfactory reproducibility and sensitivity were obtained. Due to the mentioned point this organic solvent was selected as the optimum solvent and the optimization of its volume was subjected to the next experiments. The effect of different volumes of acetonitrile within the range 0.5–6.0 mL on the desorption process of target drugs and subsequent analytical results were studied (Fig. 5d). The latter experiments showed that 1.5 mL is high enough to effectively elute the drugs from the surface of CC/ZIF-71/Fe_3_O_4_/PTh. In further experiments, it was revealed that by dividing the whole volume of desorbing agent to smaller amounts, more stable condition was achieved. Hence, in each elution, 0.5 mL of acetonitrile was used and it was repeated three times to completely desorb the target compounds during the application of ultrasonic irradiation for 3 min.

### 3.6. Influence of sample media ionic strength

It is well documented that an increase in the salt content of sample media causes a significant decrease in solubility of organic substances resulting more effective extraction yields.^51^ As a result, the effect of ionic strength on the isolation and determination of FEB and DIC was evaluated by applying NaCl as an electrolyte within the range of 0–12% w/v (Fig. 5e). Surprisingly, different results were obtained as follows: by increasing the salt amount of human plasma relatively poor extraction efficiency was obtained, which was probably due to this fact that a growth in salt concentration in aqueous media caused the viscosity of the sample to become higher leading to difficult diffusion of analytes. According to these points no salt was used in all quantification steps.

### 3.7. Reusability of CC/ZIF-71/Fe_3_O_4_/PTh

In the present experiments, after finishing the extraction process, the hybrid sorbent was washed with 2.0 mL acetonitrile and 2.0 mL distilled water during the application of ultrasonic irradiation for 7 min. Next, the CC/ZIF-71/Fe_3_O_4_/PTh was dried at room temperature (23 ± 0.5 °C) over night and applied again for further measurements of FEB and DIC. The obtained data showed that the fabricated CC modified with nanoarrays of substances is able to use about 17 times with an only 8% decrease in extraction recovery revealing notable reusability of the new designed extractor.

### 3.8. Analytical figures of merit

**Fig. 6:**
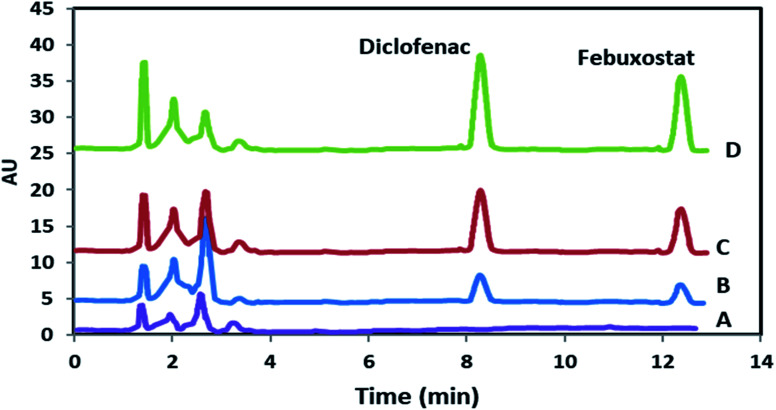
HPLC chromatograms of blank sample (A) and spiked plasma (B–D). Chromatograms of B–D refer to 50.0, 100.0 and 175.0 ng mL^−1^ of both DIC and FEB, respectively.

Analytical aspects of the developed method were evaluated using the optimized values of variables. Linear dynamic range (LDR), limit of detection (LOD), limit of quantification (LOQ), determination coefficient (*r*^2^), extraction recovery (ER) and enrichment factor (EF) as the main analytical figure of merits were investigated in detail the obtained results are summarized in Table 1. Briefly, limits of detection (LODs, *S*/*N* = 3) for DIC and FEB were 2.1 and 3.7 ng mL^−1^, respectively, furthermore, satisfactory and practical linearity (0.996≥ *r*^2^ ≥0.992) along with desirable dynamic concentration were achieved within 10–1800.0 ng mL^−1^ and 15.0–2500.0 ng mL^−1^ for DIC and FEB, respectively. Human plasma samples spiked with different amounts of target drugs as well as blank sample were subjected to the developed method and their HPLC chromatograms are shown in Fig. 6 revealing no notable matrix effect. The calibration curves of DIC and FEB were plotted before and after applying enrichment protocol and their ratios were used to calculate the value of EF. ER was estimated according to the following equation:^52^ER% = EF × (*V*_Final volume_/*V*_Initial volume of plasma_) × 100

**Table 1:** Analytical figures of merits of the USA-DMSPE-HPLC-UV for quantitation of target drugs

Analyte	LDR^a^ (ng mL^−1^)	Linear equation	(*r*^2^)^b^	LOD^c^ (ng mL^−1^)	LOQ^d^ (ng mL^−1^)	EF^e^%	ER^f^% (*n* = 3)
Diclofenac	10.0–1800.0	*Y* = 173*X* + 421	0.992	2.1	10.0	37.5	93.9
Febuxostat	15.0–2500.0	*Y* = 152*X* + 205	0.996	3.7	15.0	34.4	86.1

a Linear dynamic range (LDR).

b Determination coefficient (*r*^2^).

c Limit of detection (LOD).

d Limit of quantification (LOQ).

e Enrichment factor (EF).

f Extraction recovery (ER).

### 3.9. Precision and accuracy

Other analytical features of the current method involving intra-day and inter-day precisions as well as corresponding accuracies were examined by measuring the concentration of target drugs in the previously prepared quality control samples at amount levels of 10.0, 150.0 and 1000.0 ng mL^−1^ for DIC together with 15.0, 150.0 and 1000.0 ng mL^−1^ for FEB. In this regard, precisions based on the calculation of relative standard deviations (RSDs) of intra- and inter-day assays and accuracies based on the relative errors of target drugs were studied and related results were summarized in Table 2. The values of intra-assay (≤7.2%, *n* = 9), inter-assay (≤7.5%, *n* = 9) along with accuracy (≤8.0%) show the reliability of the presented strategy for simultaneous trace monitoring of FEB and DIC in real samples.

**Table 2:** Intra-day and inter-day accuracy and precision data in quantification of febuxostat and diclofenac in real media

Drug	Concentration (ng mL^−1^)	Intra-day, *n* = 9			Inter-day, *n* = 9		
		Found value ± SD^a^^b^ (ng mL^−1^)	RSD^c^ (%)	Accuracy^d^ (%)	Found value ± SD (ng mL-1)	RSD (%)	Accuracy (%)
Diclofenac	10.0	9.3 ± 0.5	5.4	−7.0	9.2 ± 0.6	6.5	−8.0
	150.0	140.7 ± 9.3	6.6	−6.2	159.1 ± 11.0	6.9	+6.0
	1000.0	1053.0 ± 51.4	4.9	+5.3	1066.3 ± 80.0	7.5	+6.6
Febuxostat	15.0	14.1 ± 0.9	6.4	−6.0	13.8 ± 0.8	5.8	−8.0
	150.0	157.6 ± 10.5	6.7	+5.1	139.2 ± 8.5	6.1	−7.2
	1000.0	1062.1 ± 77.5	7.2	+6.2	940.5 ± 63.4	6.7	−5.9

a Standard deviation.

b The average value of three independent measurements.

c RSD is calculated based on the following equation SD/mean value × 100.

d Accuracy is determined as (mean concentration found − known concentration)/(known concentration) × 100.

### 3.10. Application of the method for pharmacokinetic study

Eight healthy volunteers between the age of 20–40 years used the drugs under study through oral administration of a tablet containing combined dosage (100 mg diclofenac/40 mg febuxostat). The volunteer's blood were collected after 0 to 4 and 8, 12 and 24 h of taking the drug and the samples were transferred to the polypropylene tubes containing ethylenediaminetetraacetic acid and centrifuged to achieve the required human plasma, after that the samples were kept at −18 °C. All assessments were performed under the guidance of “The Committee for Research Ethics” of the Department of Pharmacy and Pharmaceutical Sciences Research Center, Tehran University of Medical Sciences. In the date of analysis, frozen samples were thawed and their deproteinization were performed as it was described before. The samples were analyzed by means of the current method and the average concentration of each drug *versus* time was drawn. In this assessment, some major pharmacokinetic features including *T*_max_, *C*_max_, AUC_0–*t*_, AUC_0–∞_, and *T*_½_ were calculated and the results were summarized in Table 3. All the obtained data show the noteworthy applicability of the recent approach for simultaneous quantification of DIC and FEB in human plasma after combination therapy.

**Table 3:** Pharmacokinetic parameters of diclofenac and febuxostat after oral administration of a fixed content combination tablet (100 mg diclofenac/40 mg febuxostat) to eight healthy volunteers^a^

Pharmacokinetic feature	Mean ± SD	
	Diclofenac	Febuxostat
*T*_max_ (h)	2.88 ± 0.30	3.15 ± 0.27
*C*_max_ (μg mL^−1^)	1.62 ± 0.45	2.13 ± 0.60
AUC_0–24_ (μg h mL^−1^)	4.47 ± 0.35	5.66 ± 0.31
AUC_0–∞_ (μg h mL^−1^)	5.07 ± 0.52	6.18 ± 0.40
*T*_½_ (h)	5.1 ± 1.1	6.3 ± 1.5

a *T*_max_: time required for reaching maximum plasma concentration. *C*_max_: maximum plasma concentration. AUC _0–24_: area under curve. AUC _0–∞_: area under curve at infinite time. *T*_½_ (h): time required for reaching to half concentration.

### 3.11. Comparison with reported methods in literature

Analytical aspects of USA-DMSPE-HPLC-UV were compared with some robust methods reported in literature to show the major advantages of the current approach in trace measurement of FEB and DIC in real samples. The results of the latter comparison are summarized in Table 4. As it is clear in this evaluation, LOD and RSD values along with dynamic range are comparable with some methods and in some cases are superior. Furthermore, most of the reported methods suffer from major drawbacks including high matrix effect, low sensitivity, the lack of simultaneous analysis ability, high extraction time, high usage of toxic materials and tedious sample preparation steps. In contrast, in the recent method, the new designed recyclable sorbent immobilized with nanoporous material offers high surface area leading to notable extraction recoveries of analyte, significant reduction in cost of analysis and lower extraction time.

**Table 4:** The comparison of USA-DMSPE-HPLC-UV with previously reported methods for quantitation of diclofenac and febuxostat on real samples^a^

Instrument	Extraction method	Extraction phase	Drug	LOD (ng mL^−1^)	LOQ (ng mL^−1^)	*r* ^2^	RSD (%)	Matrix	Ref.
RP-HPLC	SALLE	Acetonitrile/ammonium acetate	FEB	65.9	199.5^b^	0.9997	NR	Human plasma	11
RP-HPLC	LLE	Dichloromethane/isopropyl alcohol	DIC-P	9.0	25.0	0.9994	2.4 and 1.9	Human plasma	13
LC-ESI-MS/MS	LLE	Methyl *tert*-butyl ether	FEB	2.5	50.0	0.9989	NR	Human plasma	53
HPLC-DAD-FD	IL-DLLME	[BMIM][PF_6_]/[BMIM] [BF_4_]	DIC	95.0	316.0	0.9995	3	Water	54
FRET	Fluorimetric	—	FEB	210.0	638.0	0.9995	0.68 to 1.76	Human plasma	55
HPLC-UV	SBSE	PDMS	DIC	12.03	36.37	0.9999	NR	Urine	56
HPLC-UV	SPE	CTAB-coated Fe_3_O_4_	DIC	15.0	NR	0.9998	2.76 and 1.73	Human plasma/Urine	57
RP-HPLC-UV	LLE	Diethyl ether	FEB	NR	250.0^b^	NR	<15.0	Human plasma	58
HPLC-UV	USA-DMSPE	CC/ZIF-71/Fe_3_O_4_/PTh	DIC-FEB	2.1–3.7	10.0–15.0	0.992–0.996	7.2–7.5	Human plasma	This work

a RP-HPLC: reverse phase high performance liquid chromatography method. HPLC-DAD-FD: high performance liquid chromatography-diode array-fluorescence detection. LC-ESI-MS/MS: liquid chromatography-tandem mass spectrometry. FRET: Förster or fluorescence resonance energy transfer. HPLC-UV: high-performance liquid chromatography with UV detection. RP-HPLC-UV: reverse phase high performance liquid chromatography method with UV detection. SALLE: salting-out assisted liquid–liquid extraction. LLE: liquid–liquid extraction. IL-DLLME: ionic liquids-dispersive liquid–liquid microextraction. SBSE: stir bar sorptive extraction. SPE: solid phase extraction. ([BMIM][PF_6_]): 1-butyl-3-methylimidazolium hexafluorophosphate. ([BMIM][BF_4_]): 1-butyl-3-methylimidazolium tetrafluoroborate. PDMS: polydimethylsiloxane. CTAB-coated Fe_3_O_4_: cetyltrimethyl ammonium bromide-coated Fe_3_O_4_. DIC-P: diclofenac potassium. NR: not reported.

b Lower limit of quantification (LLOQ).

## 4. Concluding remarks

A four-part and recyclable magnetic sorbent was synthesized through immobilization of nano-arrays of ZIF-71/Fe_3_O_4_/PTh on the surface of carbonized cotton cloth and employed as a capable extractor for USA-DMSPE. Modification of carbonized cotton cloth with individual substances offers many advantages like high aromatic–aromatic interactions, reasonable mass transfer, tunable porosity, considerable reusability, easy-to-recycle the hybrid material from sample media using an external magnet, high surface area, *etc.* Combination of USA-DMSPE with HPLC-UV provided a practical analytical protocol for extraction and trace quantification of FEB and DIC in human plasma with desirable sensitivity and reproducibility. Furthermore, main pharmacokinetic data involving *T*_max_, *C*_max_, AUC _0–*t*_, AUC_0–∞_, and *T*_½_ were investigated to exhibit the reliability and applicability of the designed method in drug monitoring in complex matrices.

## Author contributions

All authors have agreed to their individual contributions ahead of submission.

## Conflicts of interest

There are no conflicts to declare.

## Supplementary Material
